# Commentary on MacKechnie-Guire et al. Measuring Noseband Tightness on the Lateral Aspect of the Horse’s Face. *Animals* 2015, *15*, 537

**DOI:** 10.3390/ani16030412

**Published:** 2026-01-28

**Authors:** Cathrynne Henshall, Paul McGreevy, Glenn Shea, Orla Doherty, Janne Winther Christensen, Kate Fenner, Amanda Warren-Smith, Andrew McLean

**Affiliations:** 1 School of Agricultural, Environmental and Veterinary Sciences Charles Sturt University, Wagga Wagga, NSW 2678, Australia; 2 Sydney School of Veterinary Science, Faculty of Science, University of Sydney, Sydney, NSW 2006, Australia; 3 School of Veterinary Medicine, University College Dublin, D04 W6F6 Dublin, Ireland; 4 Department Animal and Veterinary Sciences, Aarhus University, 8830 Tjele, Denmark; 5 School of Agriculture and Food Sustainability, Faculty of Science, University of Queensland, Brisbane, QLD 4343, Australia; 6 Millthorpe Equine Research Centre, Millthorpe, NSW 2798, Australia; 7 Equitation Science International, Tuerong, VIC 3915, Australia

**Keywords:** bridles, nosebands, equine welfare, ISES taper gauge, research design, equitation science, equine facial anatomy

## Abstract

The use of horses for sport is under scrutiny due to evidence that common practices such as tight nosebands may impair horse welfare. Restrictive nosebands prevent horses from performing normal comfort behaviour such as coughing and yawning. To address these concerns, the International Society for Equitation Science (ISES) developed a noseband tightness-checking device, the ISES “taper gauge,” along with a validated method that assesses how far the device can be inserted beneath the noseband at the dorsal midline of the nasal planum. However, citing concerns about the reliability of dorsal midline measurements, MacKechnie-Guire and co-authors evaluated three alternative sites: lateral to the nasal bone, the maxilla, and the mandible. They concluded that the lateral maxilla was a suitable substitute for the dorsal midline. The methods and interpretation of the findings of this study have raised concerns that measuring noseband laxity at the lateral maxilla may underestimate tightness because of the substantial volume of soft tissue at that location. This could expose horses to the welfare risks associated with overly tight nosebands. This commentary outlines the authors’ concerns and offers recommendations for how future studies might address avoid the issues raised here.

## Simple Summary

The use of horses for sport is under scrutiny due to evidence that common practices may cause harm to horses, including using equipment such as tight nosebands. Tight nosebands can prevent horses from performing normal behaviour. To test noseband tightness, the International Society for Equitation Science developed a method and a device which involved testing how far the device could be inserted under the noseband on the middle of the horse’s nose. In a recent study, MacKechnie-Guire and co-authors tested alternative sites to this accepted location and concluded that a location on the side of the horse’s face was a suitable replacement for the accepted location. The methods and interpretation of this study have raised concern that the alternative location may underestimate noseband tightness due to the anatomy of the equine head. This may expose horses to the welfare risks associated with tight nosebands. This commentary outlines the authors’ concerns along with suggestions about how future studies on this topic could be conducted to avoid the issues raised by this commentary.

## 1. Introduction

The use of horses for sport and leisure is under significant scrutiny due to increasing evidence that common practices and equipment use may impair horse welfare [1]. One such issue is noseband tightness. Nosebands are widely used in equestrian activities, and are even mandatory in some disciplines, such as dressage. Nosebands are commonly used to increase rider control by preventing the horse from performing oral behaviours such as opening their mouths to reduce bit pressures on their oral tissues [2]. While tight nosebands may give riders a competitive advantage [2], they also impair horse welfare in numerous ways. These can include contributing to the development of oral lesions [3], restricting normal behaviours like yawning and coughing [4] and long-term pathological changes to anatomical structures [5,6]. Several studies have identified a form of dose–response effect, where the tighter the noseband, the greater the risk of detriment to horse welfare [3,4]. In this context, the Federation of Veterinarians of Europe’s joint position paper [7], citing Doherty et al. [8] and Uldahl and Clayton [3], explicitly states that “equipment designed to cause pain or discomfort to modify behaviours, such as... tight nosebands for horses, should not be used”.

In response to the early findings of the aversive impacts of tight nosebands on horse welfare [9], a device was developed to facilitate reliable and repeatable noseband tightness checking in the field, the International Society for Equitation Science’s (ISES) taper gauge. This device incorporated traditional measures of tightness (a tradition metric of “two fingers”) [10,11,12,13] into a standardised tool, which when used as directed (against the dorsal midline of the nasal planum) provided a minimum amount of space under the noseband that permitted horses freedom to perform some comfort behaviours, such as mouth opening and chewing, but not all, such as yawning [4]. Despite the long availability of this knowledge as a means to ensure that competition horses are afforded this important ability to perform oral comfort behaviours while wearing bits and nosebands, research continues into noseband use that involves repeatedly exposing horses to tightness levels that severely restrict their ability to perform these behaviours and which may cause pain and discomfort [14,15]. Such research is posited as necessary to collect empirical data about the interactions between tight nosebands and equine facial tissues, such as the pressures exerted by nosebands, to inform regulatory decisions about noseband use to safeguard competition horse welfare [14]. An outcome of this research has been the development of a new FEI tool for measuring noseband tightness which provides for a reduction in the minimum amount of space under the noseband from 6.08 cm^2^ (two finger equivalent) to 4.67cm^2^ (1.5 finger equivalent) [16,17].

Given ethical concerns around deliberately exposing experimental animals to harm, particularly in the context of investigating practices which prior research has already identified as harmful, it is imperative that the rationale for such research, and its design and implementation, is clearly stated and the benefits for horse welfare robustly developed. This is particularly the case when the research is commissioned and funded by sport governing bodies for the purposes of developing regulatory policies or procedures [17] that will have a direct impact on the welfare of competition horses globally.

In order to be sufficiently robust to inform horse welfare policy changes, such research must include a suite of validated indicators and methodologies for assessing the welfare impacts of the experimental interventions on the horses. These will include behavioural and physiological measures, in addition to any other measures, such as biomechanical or pressure metrics [1]. It is imperative that the horse’s subjective experiences of the interventions are prioritised as the basis for any welfare assessment [18]. Research that makes welfare recommendations about the impacts of experimental interventions, or their analogues in real world settings without using a multimodal approach, risks failing to collect data that meaningfully reflects the intervention’s welfare impacts. Where incomplete data are then used to support reductions in minimum welfare standards (as occurred recently; [15]), horse welfare is put at risk.

In this context, this article provides commentary on a recent report ([17] the **article**) describing an equine study that used a digital calliper to determine the distance (mm) separating the surface of the skin from the inner surface of three noseband designs (cavesson, Swedish and drop). The stated rationale for the study was that the standard noseband checking site—the dorsal nasal planum—is unreliable due to variation in the underlying anatomical structures, thus necessitating the identification of alternate sites at which noseband tightness can be reliably assessed [17].

To test this, the three noseband designs were fitted to a snaffle bridle and five tightness levels (all below and none above the previous two-finger recommendation) were then established by using a modified ISES taper gauge on the dorsal midline. The five tightness levels were two fingers, 1.5 fingers, one finger, 0.5 fingers and 0 fingers. Digital calliper measurements were then taken at three locations of the head: lateral to the nasal bone, the maxilla and the mandible, to capture the distance between the noseband surface and the skin at each level of the noseband tightness. The report also includes descriptive data regarding horse behaviour during the measuring of distances at various tightness levels. However, these data were not subject to an analysis, nor was a formal ethogram of these behaviours provided. Operator preferences for each of the alternative sites was also reported. On the basis of their findings, the authors suggest the lateral maxilla is a suitable alternative to the dorsal planum for checking noseband tightness.

This study raises a number of questions regarding its rationale, methodology and conclusions. Of particular concern is the likely impact on horse welfare, should the recommendations be adopted in practice. Our commentary on the article is presented in three sections: Background (of the article); Methods and results; and Conclusions. We end with our own Summary.

## 2. Background

As previously noted, an abiding concern with regard to noseband use in equestrian sport are the known behavioural and physical harms that are caused when nosebands are tightly fastened. It is such concerns that have underpinned much of the existing research on this topic [4,9,19,20]. However, despite these existing studies, noseband impacts on horse welfare are not mentioned in the article. Given that a conclusion of this article is that sites alternative to the currently accepted dorsal nasal planum could be used for noseband checking in competition settings, this lack of assessment of the existing data in relation to horse welfare is concerning. It raises the question as to how the harms caused by tight nosebands will be additionally or equally addressed by the use of alternative checking locations compared to the nasal planum.

### Study Rationale—Anatomical Variation in the Equine Head

The rationale for conducting this study is not clearly articulated. Although it is stated that the dorsal midline is suitable for checking noseband tightness because it lacks underlying compressible soft tissues and was the site used by the representatives of the Federation Équestre Internationale (FEI) to conduct tests of a novel noseband tightness-checking device on 600 horses [17], the authors state that the dorsal midline is subject to unacceptable anatomical variation. However, no data is offered in regard to how this anatomical variation compromises the reliability or repeatability of noseband tightness-checking at the dorsal midline, such that there is an imperative to find alternative locations to address this apparent weakness. Similarly, the article does not offer evidence that the proposed alternative locations, notably the lateral maxilla (discussed in the section Methods and Results), are sufficiently free from variation so as to be a superior replacement site for the accurate assessment of noseband laxity.

Undoubtedly, there is variation in the musculoskeletal structures of the equine head that can influence the lateral profile, including the breed [21], nutritional state and sexual dimorphism [22]. However, to our knowledge and in the two studies cited by the authors in support of their assessment that anatomical variations at the nasal planum render the use of the dorsal midline unsuitable for noseband checking, limited data on this issue exist. Doherty et al. [20] reported that only one of 23 horses had an unusual and uncommon nasal profile leading to outlier readings of noseband tightness, whereas in Murray et al. [23] high repeatability and intra-horse variability was not significant and was reported for 12 horses.

That the FEI provided funding for the study is declared. However, there is no undertaking that the FEI did not have an involvement in the design of the study, an omission that is unusual in declarations of funding by an industry stakeholder.

## 3. Methods and Results

### 3.1. Choice of Noseband Type and Noseband Fitting

**Figure 1:**
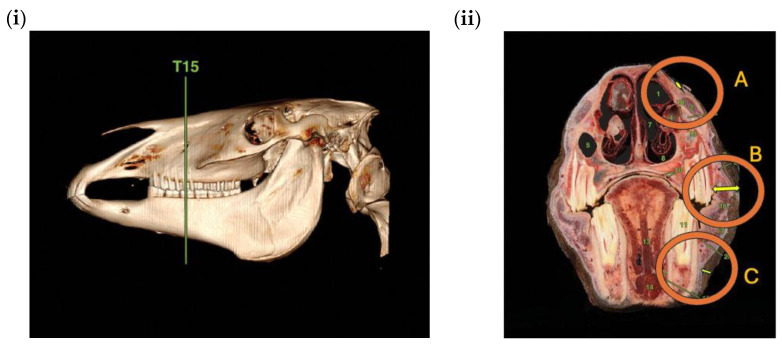
(**i**): Lateral view of equine skull where the green line indicates the location of (**ii**): transverse section of the equine head revealing clear differences in the volume of soft tissues (yellow double-headed arrows) underlying three candidate locations between the lateral nasal (A), the lateral maxilla (B) and the lateral mandible (C). Adapted from [26] Permission pending.

Of the three nosebands chosen for testing—the Swedish, cavesson and drop noseband—only the drop noseband fastens below the bit. Swedish and cavesson noseband designs are routinely used in elite dressage (in a double bridle involving two bits); however, in other contexts such as lower-level dressage, showjumping and eventing, they are commonly used with an additional strap that passes below the bit and is fitted to the curb groove (the flash). Over 40% of riders report using this design and field studies have revealed that the flash attachment may be tightened at higher tensions than the main strap of the noseband [24]. In comparison, drop nosebands are used by only between 2.3 and 10% of riders, [2,24] and given there are also a range of other designs that fasten below the bit, such as the grackle and Micklem bridle [25], the lack of inclusion of the most commonly used restrictive noseband designs limits the generalisability of the findings for the field conditions in which the alternate checking sites are proposed to be used. For example, in the case of the cavesson/Swedish design used with a flash strap, the effect of the strap is likely, depending on its tightness, to alter the position and contact of the cavesson portion against the face at the lateral maxilla and lateral nasal bone sites. Secondly, in regard to the drop noseband, they are not fitted in the same way as cavesson/Swedish nosebands. The dorsal portion of the drop noseband is positioned distal to the normal placement of the cavesson/Swedish designs. Consequently, the portions of the head the drop noseband makes contact with vary significantly from the cavesson/Swedish with flash designs. While this difference is noted by the authors to explain why the two lateral sites were not tested, the difference in the position of the dorsal portion of the drop noseband compared to the two other designs appears not to have been taken into account. Accordingly, the measurements of the drop noseband against the two other designs reported in the study cannot be directly compared. This point is further emphasised by Figure 1 from the article [17], which shows only the position of the cavesson noseband and the fact that the coloured lines added to the image to highlight the measurement locations do not extend rostrally to where the drop noseband would sit.

### 3.2. Rationale for Choice of Alternate Noseband Checking Locations

The article states that “*The first criterion for a suitable anatomical location [for noseband checking] is the presence of minimal soft tissues between the skin and the underlying bone*”. In the next paragraph, the authors state that they have “*identified three easily identifiable locations beneath the noseband on the lateral side of the skull that fulfil these requirements*”. This statement is repeated in the Discussion: “*The nasal bone, maxilla and mandible were chosen because they were easy to identify and there was minimal soft tissue intervening between the skin and underlying bone, thus providing a rigid underlying surface for the measurements*” and “*The three lateral locations used in this study were selected on the basis of being easily identified without specialized anatomical knowledge and having minimal soft tissue between the skin and underlying bone.*”

### 3.3. Existence of Soft Tissue at Preferred Alternative Measuring Site and Analysis of Underlying Anatomy

The currently accepted noseband checking site, the nasal bone, is clearly a site with minimal soft tissue between the skin and underlying bone, making its use for this purpose repeatable. This provides a reliable means to ensure that noseband tightness does not exceed acceptable or regulatory thresholds that in theory serve to protect the welfare of the horse. Consequently, if alternative sites are considered necessary, it is imperative that they are anatomically comparable, most notably in having “*minimal soft tissue between the skin and underlying bone*”. However, in this case, the premise is flawed, not least by the absence of any detail on how “minimal soft tissue” was defined, measured and confirmed.

A visual appraisal of anatomical dissection sections across these three locations (Figure 1) reveals that the authors’ assertion that the alternate sites include minimal soft tissue is not supported because there is considerable variation in underlying soft tissues between the three locations. Notably, of the three locations, the lateral maxilla (rostral to the facial crest) has the most underlying soft tissues (see Figure 1 this article). The described position is covered by a combination of buccinator (particularly if the gauge is placed only a little bit more ventral than the described location) along with the complex interdigitation of the caninus and levator nasolabialis just dorsal to the buccinator. The zygomaticus is also close to that area. If the assessor makes a small ventral movement away from this location, the assessment calliper or any assessment tool would be in the gap between maxilla and mandible, where there is an obvious deep concavity that could easily fit the device or callipers. In spite of this, the article nominates the lateral maxilla as the preferred alternative site for measuring noseband laxity.

### 3.4. Interaction of Soft Tissue and Non-Deformable Measuring Device

The study overlooks the practical effect of the compliance of voluminous soft tissues underlying the skin in the lateral maxilla location when compressed by a non-deformable measuring device. The interaction of compliant soft tissue and a non-deformable device would make reliable and repeatable noseband tightness checking at this location very difficult. Indeed, previous research [20] excluded the lateral aspect of the face as a reliable site for measuring noseband tightness because the low radius of curvature (relative flatness) at the location used in that study (defined as the location of the lateral nasal artery and vein) resulted in both much greater ease of introduction of the measuring device, and pressure readings that were one third lower than those recorded at the nasal planum. Similarly, the low radius of curvature at both the lateral nasal and lateral mandibular locations used in the current study increase the risk that an inappropriately tightened noseband could remain undetected by stewards.

### 3.5. Lack of Precision Describing Measuring Sites

The nasal bone site is simply described as “close to or over the nasomaxillary suture”. This is much less precise than the dorsal midline site to which the alternate sites are compared. Based on Figure 1 (from the article [17]) it appears that the nasal bone site was determined by being immediately caudal to the caudal extremity of the nasoincisive notch. The nasoincisive notch is readily palpable as a soft-tissue area which is not underlain by bone, associated with (lying deep to) the nasal diverticulum. The naxomaxillary suture itself is not effectively palpable in the live horse (especially if the noseband is in place over that region). The lateral maxilla site is described as “rostral to the upper edge of the facial crest”. The facial crest itself lies caudal to the position of a noseband, and the fingers/measuring device is normally inserted from a rostral to caudal direction, in other words from the opposite side of the noseband to the facial crest, the landmark being used as the reference point here.

The lateral mandible site is described as “on the lateral aspect of the mandible” Figure 1 from the article [17] implies this position is just ventral to the dorsoventral extent of the mandible at the level of the noseband. However, given there is considerable muscle cover in this region (zygomaticus, buccinator and depressor labii inferioris), it is unclear how a repeatable positioning of the gauge could be assessed. The authors state that the assessors were provided with “theoretical and practical training” prior to and on the day of the study, but whether this same combination of experience and training could be provided to everyone else in the horse industry who might be responsible for noseband checking is unclear from the descriptions of the training that were provided.

### 3.6. Further Issues

These issues are compounded by a lack of precision regarding exactly how far rostral to the facial crest the measurements were taken, as the guiding line depicting the location of the measurements in Figure 1 of the article comprises approximately ¼ of the length of the profile. This lack of precision is significant, because it renders the study unrepeatable and means that the reliability and repeatability of any measuring device designed to be used in this location is likely to be poor. In addition, in contrast to the dorsal midline, none of these alternative sites have anatomical structures on the side that the gauge is inserted from that are immediately recognisable for lay personnel, making them harder to locate precisely, reducing the repeatability and reliability of measurements taken in the field at these locations.

### 3.7. Behavioural Data

The article reports on anecdotal accounts of horses performing undesirable responses during noseband checks in this study and elsewhere. However, it refers to these instances only in the context of the ISES taper gauge and does not compare the frequency of such behaviours with other similar testing examples, such as the testing of the FEI device which is also designed to be used on the dorsal midline and which has been tested on 600 horses (p. 14) [17]. No data regarding the behavioural responses of horses during these tests have been provided which would provide additional context to the implication that the noseband checking at the dorsal midline induces behavioural indicators of negative affect and that, consequently, alternative locations for noseband checking are warranted.

In discussing the horse’s responses to the use of both the insertion of the ISES tape gauge and the calliper insertion at the three locations, the article reports descriptive data in relation to the subjects’ behavioural responses and notes that there were fewer reported responses to the calliper use at the lateral maxilla. However, there is no explanation of the sampling methodology employed (whether via video recording or contemporaneous note taking), nor which behavioural responses the horses showed in relation to each of the individual events (insertion of tape gauge or callipers), their frequency in relation to the individual tightness levels and nor is an ethogram of the behaviours included in the report. “Aversive” responses to the calliper use are referred to; however, the specific behaviours deemed aversive are not described. Collectively, the lack of these data substantially limits the repeatability of the study. In describing both the behavioural responses to the noseband checking and calliper use and the cumulative withdrawal of over 10% of subjects either due to unacceptable behavioural responses or owner concern as noseband tightness increased [17], the authors have provided incidental evidence that nosebands, even at high laxities, may induce negative affect in horses.

### 3.8. Receiver-Operator Characteristic

The article assesses the use of hand-held callipers at the three locations by the seven operators and the resultant coefficients of variance between operators to categorise the reliability attributes of testing for space under the noseband at the 1.5 and 2.0 finger equivalent tightness levels. The study found ‘good’ agreement (among tests) with the maxillary site but only ‘acceptable’ agreement with the mandibular site at the two tightness levels [17]. The existing location (dorsal midline) was not tested with the callipers, a missed opportunity to include a control. If the objective of the study was to improve the repeatability and reliability of noseband testing as suggested, the alternative checking method (the digital calliper) should have also been tested at the existing site. Although the paper does include an extensive discussion of the ROC data in relation to the three alternate sites, it appears that it is ultimately the operators’ preferences that inform the selection of the lateral maxilla as the replacement site for noseband checking. However, information is not provided in regard to how this preference data was collected, such as by survey instrument or informal discussion.

In addition, while the researchers were blinded to the measurements (from the digital gauge) we are not informed as to whether or not the operators were blinded to the intended purpose of the study. This is important when one considers the operator effect(s) that may have influenced how the gauge was manually introduced into the spaces as they were assessed. This limitation is acknowledged but the relevance of operator variability as an influence on operators’ reported preference to the conclusions is not discussed.

### 3.9. Differences in Facial Contact Between Cavesson and Swedish Nosebands

The article reveals important differences between cavesson and Swedish nosebands. It notes critical differences in the malleability of the Swedish noseband and suggests that these differences are of practical importance. Independent of noseband type, the materials used to construct nosebands and the properties of any padding influence the malleability of all nosebands. This malleability renders a distance measurement at the location preferred by operators (the lateral maxilla with its underlying soft tissue) of little value in assessing the consequences for horse welfare. Welfare assessments require the use of horse-based indicators, such as behaviour, pathology and sub-noseband pressures. Furthermore, as this article notes, the padding in Swedish nosebands may make the mandibular location for noseband checking purposes problematic. It then notes that for “*the cavesson noseband at the 1.0 and 0.5 finger equivalent adjustments, the maxilla had greater noseband separation than the lateral nasal site. However, the magnitude of the difference was <1.05 mm, which seems unlikely to be biologically significant.*” This interpretation of this data point is troubling if considered from the perspective of horse welfare and horse’s subjective experiences of equipment use. It could be viewed as a reductionist interpretation to state that 1 mm difference in a calliper measurement at a single site of measurement represents a non-biologically significant difference for horses, given that, as the authors have noted extensively elsewhere in the paper, there are significant differences in skin separation distances and indeed pressures under the noseband between 1 and 1.5 finger equivalents at other sites, [14,15] and that noseband padding thickness and malleability may vary depending on the noseband design and materials. These factors emphasise the necessity of including behavioural measures, such as post-inhibitory rebound (e.g., Fenner et al. [4]) in studies and that equipment is tested in real-world conditions reflecting the pressures horses in industry routinely experience.

## 4. Limitations

### 4.1. Lack of Generalisability of Findings for In-Field Settings

The study was conducted only on horses wearing snaffle bits and therefore its results are not necessarily relevant to horses wearing a double bridle, notably all horses competing in elite dressage. Given that a rationale for the study was to identify alternate locations for noseband checking in the field, the lack of testing of horses wearing a double bridle substantially limits the generalisability of the findings. In the context of field checking at elite dressage competitions, the interaction of the configuration of the double bridle and the impact of the two bits on the horse’s tongue and surrounding soft tissues could substantially decrease the utility of the preferred testing site (lateral maxilla). The configuration of a double bridle, including the effects of the curb attaching to the cheekpiece, bridoon and bridoon strap would likely complicate access to the lateral maxilla, increasing the risk of operator error and a lack of consistency. Secondly, as the equine tongue is a hydrostat [27] that maintains a given volume within the mouth, the volume and weight occupied by two bits in the oral cavity will impact the relative shape taken by the tongue relative to the hard and soft tissues in and around the oral cavity. Consequently, depending on the size of the bits, bulging cheeks may be seen lateral to the maxilla in horses wearing a double bridle and this phenomenon is likely to interfere with reliable and repeatable checking of noseband tightness at the lateral maxilla.

### 4.2. Utility of Lateral Maxilla

Ultimately, of the three locations, the lateral maxilla was the location preferred (by the operators) in 80% of the studied horses. This appears to reflect the ease with which measurements could be taken. Logically, this relative ease could be a direct result of the extra compliance that this location offers because of the underlying soft tissues, as noted above. Furthermore, it is not clear how practical assessment of tightness at the lateral maxilla avoids artefacts created by adjacent noseband padding that serve to lift the noseband away from the skin at the lateral maxilla. Interestingly, the cavesson noseband in the study had 4 mm of padding, but the authors acknowledge that padding is especially abundant in Swedish nosebands. This noseband design is favoured among riders competing in dressage, the discipline noted for the tightest nosebands [20]. Prior to the nomination of lateral maxilla (“rostral to the facial crest”) as the preferred alternative candidate site for assessing noseband tightness, the lateral nasal and lateral maxilla were also identified as suitable sites that may provide an “addition to dorsal midline measurements”. However, it is not clear *when* additions to dorsal midline measurement are needed.

### 4.3. Noseband Design Differences

In addition, the authors note that there were significant differences in the separation of the cavesson noseband from the skin among all noseband tightness levels, but that with the Swedish noseband (with its additional padding) many of these differences (most notably between 1.5 fingers and two fingers) could not be detected. This is important because, in MacKechnie-Guire et al. (2024) [14], there was no significant difference in sub-noseband pressures between 1.5 fingers and two fingers’ space. As noted above, on that basis, they argued for the use of the tighter setting (1.5 fingers) for maximum noseband tightness and this non-significant finding supported use of the new FEI measuring device, providing less area in cross-section under the noseband than the ISES taper gauge allows. Given that, as noted in the article [17], Swedish noseband padding designs vary, not least in the stiffness of their padding and its relative malleability, it is disappointing that the current non-significant findings and those of the same group’s earlier report [14]) have been used to justify the choice of the higher tightness level for the purposes of safeguarding horse welfare.

### 4.4. Behavioural Responses to Noseband Checking

It is suggested that the behavioural responses shown by the horses to noseband checking in this study may have been due to repeated introduction of the ISES taper gauge [17]. However, given the well-recognised ability of horses to habituate to new handling procedures that are implemented according to learning theory [28] it is plausible that the horses were responding to the aversive impacts of the interaction between the taper gauge insertion and the increasingly tighter nosebands they were exposed to. As there is a lack of data about what aspects of the taper gauge use elicited the behaviours, this cannot be verified. The accumulated withdrawal of horses from the study as noseband tightness levels increased due to undesirable behavioural responses points to the aversive characteristics of nosebands that encircle the jaw, even in the relatively benign conditions of this study. This could function as a proxy welfare indicator, and the study would have benefited from a more detailed discussion of what characteristics of the noseband tightness or the checking process likely elicited the behavioural responses. The authors acknowledge that the response of horses to noseband tightness checking was individual and suggest that riders ‘*consider their horses’ behavioural responses in determining an appropriate noseband setting*’, but they do not refer either to their own reported behavioural indicators or to the substantial body of research already carried out on this topic as a possible appropriate source of guidance.

## 5. Conclusions and Wider Concerns About the Effect of Research to Impair Horse Welfare

When assessing the impact of equipment use on horses and their welfare, their subjective experience of horses is a critical factor [18]. This is particularly so where such research findings will be used to inform regulatory decisions that will ultimately impact thousands of animals in field/competition settings. Research focussed on the biomechanics of equipment which fails to take into account the subjective experience of the horse cannot be used to infer welfare impacts. Given that the findings of this study have been cited as a basis for making changes to accepted procedures, the failure to assess welfare indicators or consider the means by which the recommendations could impact horses in field settings means that, rather than supporting regulatory procedures to protect animal welfare, the narrowly defined findings are used to support changes that could *impair* welfare because welfare indicators are not assessed. An abiding question is why, in the context of safeguarding horse welfare, there is a need for ongoing research into noseband tightness levels that will, presumably, be prohibited now that new regulations mandating a minimum noseband laxity at 1.5 finger tightness are enacted [16].

In this vein there is a question as to the basis of the rationale to add and test the 1.5 finger increment to the long-standing ISES taper gauge device which in its original form, provided a repeatable means to ensure that horses were given a minimum of two-finger space under the noseband. In the light of ongoing concerns about the impact of equipment on horse welfare, the traditional recommendation of a higher minimum space allowance and the existing means to assess compliance with this minimum allowance, it is unclear why it was thought necessary to study the effects of *less* space on horses, given the existing body of evidence.

While we acknowledge that the investigation of factors relating to the fitting and checking of equipment used on horses is welcome, given that the authors make recommendations about the potential benefits of the preferred alternative location they identify (lateral maxilla), the lack of a genuine rationale for the benefits to horse welfare of this location is a concern. As noted above, this weakness is compounded when one appreciates that the alternative locations will be subject to the same apparent variability as the dorsal midline. Additionally, if the lateral maxilla was adopted as the preferred noseband checking site, this may lead to significant compromises to horse welfare due to the risk that noseband checking in this location will fail to adequately assess noseband tightness.

This report appears to accompany the two previous studies from the same research group (MacKechnie-Guire et al., 2024 [14]; Clayton et al., 2024 [15]) that have adopted similar approaches to argue, on the basis of non-significant findings arising from small sample sizes, for a 24% reduction in the space under the noseband. These findings have been effectively used to endorse a reduction in the space available for jaw movement beyond the limit set by the current ISES taper gauge, a tool used widely for the past decade (e.g., Doherty et al., 2017 [20]) and the standard instrument selected for the current study. We have addressed our concerns about those studies in the journal that published them [29,30].

Recently developed guidelines now provide researchers in animal behaviour modification and human–animal interaction with a comprehensive framework for incorporating animal-based welfare indicators, including species-specific indicators of subjective states, into research protocols [31]. The future application of the COMPASS Guidelines in human–animal research designs, such as the study critiqued here, will help address the concerns raised and ensure that proposed welfare implications are grounded in a holistic, animal-centred understanding of welfare.

Our concern with this study is that, based on its limited consideration of the welfare implications of changing the noseband checking site, regulatory bodies may use the current study to justify checking noseband tightness on the lateral side of the face without any acknowledgement of the considerable differences in the soft tissues that lie under the skin in this location. The omissions in this study may encourage international horse sports regulators to check for noseband tightness in this highly compliant region, thus allowing nosebands to be tighter than is currently considered acceptable. If this occurs, such a development would represent a retrograde step in horse welfare at a time when horse sports need to demonstrate progress in improving horse welfare. 

## Data Availability

No new data were created or analyzed in this study.
